# Factors Influencing Drug Uptake during Mass Drug Administration for Control of Lymphatic Filariasis in Rural and Urban Tanzania

**DOI:** 10.1371/journal.pone.0109316

**Published:** 2014-10-08

**Authors:** William J. Kisoka, Paul E. Simonsen, Mwelecele N. Malecela, Britt P. Tersbøl, Declare L. Mushi, Dan W. Meyrowitsch

**Affiliations:** 1 National Institute for Medical Research, Dar es Salaam, Tanzania; 2 Department of Veterinary Disease Biology, University of Copenhagen, Copenhagen, Denmark; 3 Department of International Health, Immunology and Microbiology, University of Copenhagen, Copenhagen, Denmark; 4 Tumaini University, Kilimanjaro Christian Medical University College, Moshi, Tanzania; 5 Department of Public Health, University of Copenhagen, Copenhagen, Denmark; University of Illinois, United States of America

## Abstract

**Background:**

In most countries of Sub-Saharan Africa, control of lymphatic filariasis (LF) is based on annual mass drug administration (MDA) with a combination of ivermectin and albendazole. Treatment coverages are however often suboptimal for programmes to reach the goal of transmission interruption within reasonable time. The present study aimed to identify predictors and barriers to individual drug uptake during MDA implementation by the National LF Elimination Programme in Tanzania.

**Methods:**

A questionnaire based cross sectional household survey was carried out in two rural and two urban districts in Lindi and Morogoro regions shortly after the 2011 MDA. 3279 adults (≥15 years) were interviewed about personal characteristics, socio-economic status, MDA drug uptake among themselves and their children, reasons for taking/not taking drugs, and participation in previous MDA activities for LF control.

**Findings:**

The overall drug uptake rate was 55.1% (range of 44.5–75.6% between districts). There was no overall major difference between children (54.8%) and adults (55.2%) or between females (54.9%) and males (55.8%), but the role of these and other predictors varied to some extent between study sites. Major overall predictors of drug uptake among the interviewed adults were increasing age and history of previous drug uptake. Being absent from home during drug distribution was the main reason for not taking the drugs (50.2%) followed by clinical contraindications to treatment (10.8%), missing household visits of drug distributors (10.6%), and households not being informed about the distribution (9.0%).

**Conclusion:**

Drug uptake relied more on easily modifiable provider-related factors than on individual perceptions and practices in the target population. Limited investments in appropriate timing, dissemination of accurate timing information to recipients and motivation of drug distributors to visit all households (repeatedly when residents are absent) are likely to have considerable potential for increasing drug uptake, in support of successful LF transmission elimination.

## Introduction

Lymphatic Filariasis (LF) is a mosquito transmitted parasitic disease which in Africa is caused by the filarial nematode *Wuchereria bancrofti*. Although LF is not associated with significant mortality, its attendant debilitating ‘acute attacks’ and disfiguring chronic manifestations (primarily hydrocoele, lymphoedema and elephantiasis) cause suffering and social stigma to the affected individuals and impedes economic performance [1]–[3]. Globally, LF affects an estimated 120 million people, out of whom 44% are in Sub-Saharan Africa, and LF has been ranked as one of the world's leading causes of permanent and long-term disability [4], [5]. The major burden of LF is found in rural areas, but it is also endemic in less developed peri-urban and urban areas [6].

In 1997, the World Health Assembly passed a resolution calling for global elimination of LF as a public health problem. The World Health Organization subsequently launched the Global Programme to Eliminate Lymphatic Filariasis (GPELF) with the major goal to eliminate LF as a public health problem by the year 2020 [7]. The programme has a twofold aim of interrupting transmission by annual mass drug administration (MDA) and alleviating suffering and disability by applying measures for morbidity control. MDA is based on annual distribution of a single dose of albendazole in combination with either diethylcarbamazine or ivermectin to all eligible individuals, with the main purpose to kill circulating microfilarae produced by the adult worms, and thereby reduce the level of transmission in the endemic communities. In addition to eliminate the microfilariae from the blood, these drug combinations have beneficial effects by reducing the burdens of intestinal helminths and ectoparasites in those treated [4], [8]. To achieve the goal of elimination of LF as a public health problem, it is crucial that a major proportion in the target community adhere to treatment and take the offered tablets once a year for a period of 5 to 6 years [4], [9], which is believed to correspond to the reproductive lifespan of the adult parasitic worms. A minimum effective population drug uptake rate is considered to be 65% [10]. However, there are many challenges in reaching such high coverage [11], and studies from LF control programmes in different parts of the world indicate that drug uptake rates are often suboptimal [12]–[18].

LF is widespread in Tanzania [19], and especially the coastal areas and areas around the great lakes are characterized by high levels of infection and disease [20]–[24]. It is estimated that over 34 million individuals live in endemic foci in Tanzania and that 5-6 million individuals are affected by one or more clinical manifestations of LF [25]. The National Lymphatic Filariasis Elimination Programme (NLFEP) was launched in Tanzania in 2000, and the first MDA with albendazole and ivermectin was implemented in endemic areas of Coast Region near Dar es Salaam in the same year. In 2009, the NLFEP was integrated in the Neglected Tropical Disease Control Programme (NTDCP), which also conducts MDA for other neglected tropical diseases. The MDA activities have gradually expanded, and now cover 17 of the 25 regions in Tanzania Mainland.

Results of previous cross-sectional surveys with focus on MDA activities in Tanzania have reported suboptimal drug coverage rates within the range of 31–62% [26]–[28]. However, the absence of detailed information on both programme/provider related factors and individual predictors for drug uptake is presently a main barrier for an informed discussion on how to improve and optimize MDA strategies in order to increase overall drug coverage rates. The present study comprises a component of a larger Tanzanian research project with the main aim of describing and analyzing MDA activities in order to develop improved programme strategies for control of LF and other neglected parasitic infections. The overall objective of the present study was to assess, through household questionnaires, the associations between selected predictors and individual drug uptake shortly after the implementation of MDA in two rural and two urban districts in Tanzania.

## Methods

### Study areas

**Figure 1:**
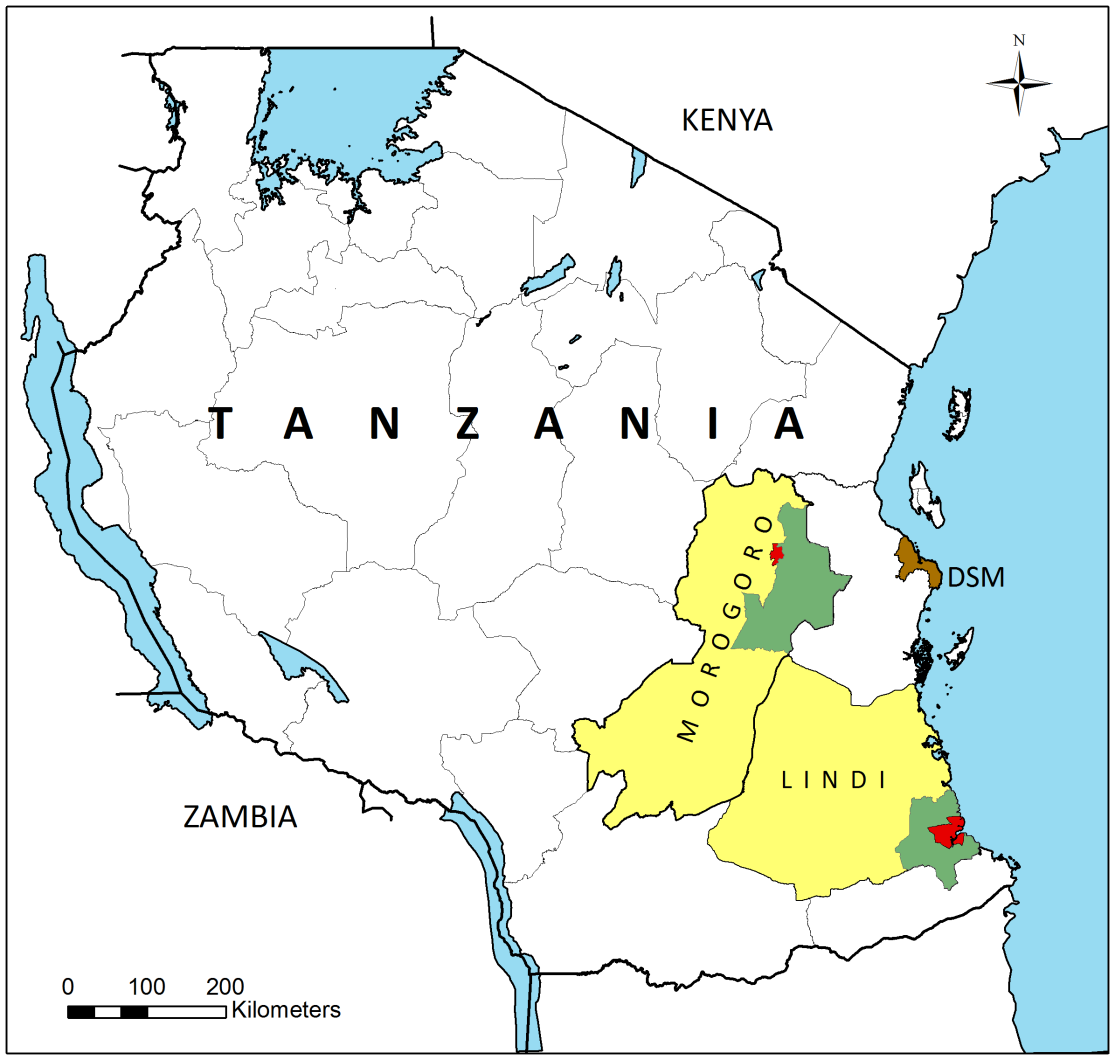
Map showing the location of the study sites in Lindi and Morogoro Region, Tanzania. Red  =  the two urban study districts; Green  =  the two rural study districts; Yellow  =  remaining parts of the two study regions; DSM  =  Dar es Salaam.

The study was carried out in Lindi and Morogoro regions, Tanzania (Figure 1). From both regions a rural and an urban district were included. The selected urban districts were those enclosing the regional capitals (Lindi and Morogoro towns), whereas the rural districts were purposively selected among those neighboring the urban district.

Lindi Region is located along the Indian Ocean coast in south-eastern Tanzania (Figure 1). The region is divided into six districts. Among these, Lindi Municipality (in the following called Lindi Urban) and Lindi Rural were included in the study, which took place in May 2011. Lindi Urban is about 470 km south of Dar es Salaam, and administratively it is divided into 13 wards. Six wards (4 central and 2 peri-urban; Rahaleo, Matopeni, Nachingwea, Mwenge, Mtanda and Msinjahili, respectively) with a total population of 23,747 were selected for the study. Lindi Rural is surrounding Lindi Urban to the North, West and South and is divided into 28 wards. The main ethnic groups in the district are the Yao, Mwera and Makonde. Agriculture forms the mainstay of economic activities in the district, whereas small-scale sea-fishing is practiced among those living along the sea. One ward (Nachunyu), with a population of 9713 and located approximately 75 km south of Lindi town, was selected for the study.

Morogoro Region is located more inland, in the eastern/central part of Tanzania (Figure 1), and is divided into 7 districts. Among these, Morogoro Municipality (in the following called Morogoro Urban) and Morogoro Rural were included in the study, which took place in August 2011. Morogoro Urban is located at the base of the Uluguru Mountains about 210 km to the east of Dar es Salaam and is divided into 19 wards. Three wards (two central and one peri-urban; Kingo, Kichangani and Kingolwira, respectively) with a total population of 44,063 were selected for the study. Morogoro Rural is located in the north-eastern part of Morogoro Region and is divided into 25 wards. The main ethnic groups in the district are Luguru, Kutu and Zigua, but pastoral Masai and Sukuma are also common. The majority of inhabitants are engaged in farming, growing both subsistence and cash crops, while other activities include fishing, forestry and small scale business. One ward (Mngazi), with a population of 9,528 and located approximately 120 km south of Morogoro town was selected for the study.

### LF control activities in the study areas

The National Lymphatic Filariasis Elimination Programme (NLFEP) in Tanzania is administering MDA with a combination of ivermectin (150–200 µg/kg body weight) and albendazole (400 mg) to individuals aged ≥5 years in LF endemic areas. After integration of the NLFEP in the Neglected Tropical Diseases Control Programme (NTDCP) in 2009, the MDA activities for LF were combined with mass treatment programmes for other neglected tropical diseases (NTDs). For the districts included in the present study the other activities include annual school based praziquantel treatment for schistosomiasis and annual community based treatment with anzithromycin for trachoma (the last activity not in Lindi Urban), usually implemented two weeks after the MDA for LF. In principle, MDA for LF is carried out as community directed treatment, with the drug distributors being selected by the general community. However, in some cases drug distributors are selected by village leaders or health personnel from nearby health facilities.

MDA for control of LF was implemented in Lindi Region (including the present study areas) in 2003, 2004 and 2005 after which the activities stopped due to financial constraints and the consolidation into the integrated NTDCP. The MDA for LF in May 2011 (prior to the present study) was therefore carried out after a period of 6 years without MDAs.

In parts of Morogoro Region, Community Directed Treatment with Ivermectin (CDTI) for control of onchocerciasis has been carried out since 1997. Morogoro Rural was covered by this activity in 2004 and 2006 only whereas Morogoro Urban was not included at all. MDA for control of LF started in 2007 in Morogoro Rural and in 2009 in Morogoro Urban, as part of the expanded mandate of the African Programme of Onchocerciasis Control (APOC) to NTDs, and has been implemented annually since then. The MDAs for LF in July 2011 (prior to the present study) were thus rounds 5 and 3 in these districts, respectively.

### Study design

The study was cross-sectional and questionnaire-based with a main focus on self-reported information on drug uptake shortly after the 2011 rounds of MDA for LF. The investigators and interviewers were not associated with the MDA staff or engaged in MDA activities. Clusters of households were prior to implementation of MDA activities randomly selected in all four study sites using a multi-stage cluster-based sampling strategy. Results of a sample size calculation indicated that in each of the four study sites an appropriate sample of 980 individuals aged ≥15 years was needed. However, a large number of individuals had to be excluded after data had been gathered, specifically from Morogoro Rural, since they had been interviewed prior to the drug distribution. Despite the exclusions we did not encounter results of statistical analysis, which suggest lack of power in our analyses.

For the selected study wards in both districts of Lindi Region, the treatment registers prepared by the NTD Control Programme for the 2011 MDA for LF were used for random selection of households to be included in the present study. For both study districts in Morogoro Region, the treatment registers prepared by the NTD Control Programme for the 2011 MDA had not been properly updated and therefore were unsuitable for the present study. Instead, a number of neighborhoods/hamlets (administrative units below the ward level in urban and rural areas, respectively) were randomly selected from the study wards, and all households (and their inhabitants) in these units were registered during house to house visits. Households were thereafter randomly selected for the study. From all four districts, households were selected to give a study population of approximately 1000 individuals aged ≥15 years. It was later realized that a proportion of the selected households in Morogoro Rural had been interviewed for the present study prior to drug distribution. Therefore, all inhabitants from this study site who said their household had not been offered drugs were subsequently excluded from the study.

### Questionnaire and interviews

The questionnaire included questions on age, gender, socio-economic indicators (educational status, ownership of household items, household ownership status), religion, drug intake during the 2011 MDA round, reasons for taking/not taking drugs, and participation in previous MDA activities for LF control. The questionnaire was initially developed in English, translated into Swahili and subsequently back-translated to English for validity purposes. The questionnaire was pilot-tested in Morogoro Region and subsequently revised.

In all four study areas, the interviews were carried out 3–9 days after distribution of drugs. In each visited household, individuals aged ≥15 years were approached and requested to participate in the interview after the interviewers had explained the outline and purpose of the research and asked for their consent. In order to include all registered members of a household, the interviewers made up to three visits to the same household. During the first visit, the interviewer also collected data on the number, age and sex of children aged 5–14 in the household, and whether they had taken the drugs during the MDA.

### Data analysis

Double entry of data and subsequent quality control of entries and data analysis were carried out using EpiData (version 3.1) and SPSS 20 (IBM version 20.0), respectively (Table S1-S4). During data analysis, individuals were divided into three age groups (15–29 years, 30–49 years and ≥50 years). Educational status was also divided into three groups: Primary education not completed, primary but not secondary education completed, and secondary education completed. Individual wealth scores were calculated based on whether at least one household member owned a radio (2 points), a bicycle (4 points), a television (6 points), a motorcycle (8 points) or a car (10 points). The sum of points was calculated and categorized into a low (0 points), medium (2–6 points) or high (≥7 points) wealth index for the individual.

Groups were compared statistically by Chi-square tests and oneway ANOVA, as appropriate. Bivariate and multivariate analyses were used to calculate strength of statistical associations presented as odds ratios (ORs) between predictors and self-reported drug uptake. Since the number of interviewed individuals varied between the households, a generalized estimating equation (GEE) model was used to compensate for a possible cluster effect of drug uptake in households and at the same time adjust the associations for the confounding effects of other co-variables.

### Ethical statement

All individuals were asked to give informed verbal consent prior to interviews, and parents/guardians were asked for permission to interview individuals <18 years. Their verbal consent to participate and/or to allow their minors to participate was recorded in each questionnaire form. Verbal consent is the traditional way for making agreements in the study areas, whereas written consent is unfamiliar and would cause distrust of true intentions and refusal to participate. Research and ethical clearance for the study (including the use of verbal informed consent) was provided by the Medical Research Coordinating Committee of the National Institute for Medical Research, Tanzania (reference number NIMR/HQ/R.8a/Vol. IX/1073).

## Results

### Study population

The initial selection provided a total eligible population of 4053 adult individuals aged ≥15 years from the 4 districts combined, and 4003 (98.8%) of these were interviewed. However, 350 individuals subsequently had to be excluded due to incomplete or inconsistent data (age, gender, drug uptake data missing; or contradictions between data on drug uptake and drug related statements), and 374 individuals from Morogoro Rural had to be excluded because interviews were performed prior to drug distribution. Hence, the total valid study sample included in the analyses was 3279 adults (80.9% of the eligible population) from 747 households. Among these, 812, 1123, 429 and 915 were from Lindi Rural, Lindi Urban, Morogoro Rural and Morogoro Urban, respectively (Table 1). Overall, the mean age was 36.9 years, there were more females (64.2%) than males (35.8%), there were more Muslims (72.2%) than Christians (26.5%), and these characteristics differed significantly between the four districts (Table 1).

**Table 1:** Characteristics of the interviewed adult study populations and their children from the four study sites in Lindi and Morogoro Region, and the reported drug uptake rates.

	Lindi Rural	Lindi Urban	Morogoro Rural	Morogoro Urban	Total	P-value
Interviewed adults (≥15 years)						
No. adults	812	1123	429	915	3279	-
Female: male ratio	1.45	2.26	1.25	1.98	1.80	<0.001*
Mean age in years	37.3	37.2	38.7	35.2	36.9	<0.001**
No. households	164	226	162	195	747	-
Mean no. individuals/household (range)	4.89 (1–17)	4.94 (1–12)	2.64 (1–6)	4.66 (1–19)	4.76 (1–19)	-
Muslim: Christian ratio	15.8	5.3	1.1	1.0	2.7	<0.001*
No. of adults taking the drugs (%)	379 (46.7)	497 (44.3)	262 (61.1)	672 (73.4)	1810 (55.2)	<0.001*
Children from same households (5–14 years)						
No. children	455	679	259	549	1942	-
Female: male ratio	0.94	0.93	1.02	0.79	0.90	NS*
Mean age in years	8.7	9.4	9.4	9.6	9.3	<0.001**
No. of children taking the drugs (%)	200 (44.0)	304 (44.8)	126 (48.6)	435 (79.2)	1065 (54.8)	<0.001*
Adults and children combined						
No. individuals	1267	1802	688	1464	5221	-
No. of individuals taking the drugs (%)	579 (45.7)	801 (44.5)	358 (52.0)	1107 (75.6)	2875 (55.1)	<0.001*

*) Chi-square test.

**) Oneway ANOVA.

In addition to the interviewed adult population, information about drug uptake was obtained from parents/caretakers for a total of 1942 children aged 5–14 years (Table 1). Of these, 922 (47.5%) were girls and 1020 (52.5%) were boys, and their mean age was 9.3 years.

### Drug uptake

The drug uptake rates among the interviewed adults and the children from the same households in the four districts are shown in Table 1. The overall drug uptake rate for all individuals (adults and children) and all four districts combined was 55.1%. Overall, the rate was higher in Morogoro Region (68.1%) than in Lindi Region (45.0%). The highest and lowest rates were observed in Morogoro Urban (75.6%) and Lindi Urban (44.5%), respectively. In three of the four districts there were no major differences in drug uptake rates between children and adults. However, in Morogoro Rural, the drug uptake rate was considerably higher in the adults (61.1%) than in the children (48.6%).

The drug uptake rates among the interviewed adults by selected variables for each of the four districts are presented in Table 2. Whereas Morogoro Rural had significantly higher drug uptake rates for females than for males, the opposite was the case for Morogoro Urban. No significant differences were seen between females and males in the two districts of Lindi. Drug uptake rates increased significantly with increasing age in three of the districts, whereas this was not the case in Morogoro Rural (Table 2). In both districts of Morogoro, there was a significant trend of higher drug uptake rates among those who had completed primary school than among those who had not, but such trend was not seen in the two Lindi districts. With the exception of Morogoro Urban, where individuals with medium-high wealth index had significantly higher drug uptake rates than those with a low wealth index, drug uptake rates showed no relation to wealth index in the other three districts. In both districts of Morogoro, individuals who stayed in rented households had higher drug uptake rates than those who stayed in households with another status (very few or none stayed in rented households in the two Lindi districts). In Morogoro Urban, there was a significantly higher drug uptake rate among Muslims than among Christians, whereas drug uptake rates showed no relation to religion in any of the other three districts. The most persistent and statistically significant trend in drug uptake rates, which was observed across all four districts, was the relationship to previous history of drug uptake. In all four districts the drug uptake rate increased with number of previous times the individuals had participated in MDA. In e.g. Morogoro Urban the drug uptake rate among individuals who had not previously taken drugs was 46.9% but it increased to 95.7% among individuals who had taken drugs three times or more prior to the current MDA.

**Table 2:** Drug uptake rates in relation to personal characteristics among the interviewed adult study populations from the four study sites in Lindi and Morogoro Region.

Characteristic	Lindi Rural		Lindi Urban		Morogoro Rural		Morogoro Urban	
	No. individuals (% drug uptake)	P-value	No. individuals (% drug uptake)	P-value	No. individuals (% drug uptake)	P-value	No. individuals (% drug uptake)	P-value
Gender (n = 3279)								
Female	331 (48.6)	NS	344 (45.9)	NS	191 (67.0)	0.024	307 (67.4)	0.003
Male	481 (45.3)		779 (43.5)		238 (56.3)		608 (76.5)	
Age group (n = 3279)								
15–29 years	318 (37.4)	<0.001	422 (38.6)	0.002	152 (61.2)	NS	386 (67.1)	0.001
30–49 years	319 (52.4)		476 (45.2)		174 (60.3)		376 (77.9)	
≥50 years	175 (53.1)		225 (52.9)		103 (62.1)		153 (78.4)	
Level of education (n = 3006)								
Not completed primary school	209 (53.6)	NS	116 (47.4)	NS	106 (51.9)	0.024	64 (71.9)	0.025
Completed primary school	458 (48.5)		871 (44.2)		321 (64.2)		744 (75.4)	
Completed secondary school	2 (50.0)		25 (64.0)		0 (0.0)		90 (62.2)	
Wealth index (n = 2750)								
Low	167 (46.1)	NS	321 (45.2)	NS	125 (74.4)	NS	45 (62.2)	0.027
Medium	608 (46.5)		564 (45.2)		190 (63.7)		190 (80.5)	
High	18 (66.7)		204 (40.7)		8 (62.5)		310 (78.4)	
Home ownership status (n = 3224)								
Own	537 (46.9)	NS	401 (43.6)	NS	307 (63.5)	<0.001	253 (72.7)	0.003
Rented	0 (0.0)		3 (66.7)		42 (78.6)		385 (78.2)	
Friends/relatives	237 (45.6)		404 (45.3)		67 (43.3)		240 (65.0)	
Employer	35 (51.4)		299 (43.8)		2 (0.0)		12 (83.3)	
Religion (n = 3260)								
Muslim	760 (46.3)	NS	935 (44.1)	NS	216 (63.0)	NS	455 (78.5)	0.002
Christian	48 (52.1)		178 (46.1)		199 (60.3)		439 (68.1)	
Other	3 (66.7)		7 (14.3)		12 (41.7)		8 (62.5)	
Previous MDA drug uptake (n = 3168)								
Not before	233 (33.0)	<0.001	453 (40.0)	<0.001	75 (21.3)	<0.001	262 (46.9)	<0.001
Once	215 (50.2)		317 (42.0)		144 (68.8)		361 (83.4)	
Twice	256 (53.5)		234 (51.7)		115 (67.0)		227 (83.7)	
Three or more times	67 (67.2)		71 (63.4)		91 (74.7)		47 (95.7)	

Results of bivariate and multivariate analysis of predictors for drug uptake among the interviewed adults from all four study sites combined are shown in Table 3. After adjusting for confounding and the potential cluster effect of household exposure, the results of the GEE model indicated that age group, renting a house and history of previous drug uptake remained as statistically significant predictors for drug uptake. Hence, individuals in the age groups 30–49 years or ≥50 years were approximately 30–40% more likely to take drugs as compared to individuals in the age group 15–29 years. To live in a rented house or apartment as compared to being an owner of a house or apartment more than doubled the chance of taking drugs. The strongest statistically significant predictor for drug uptake was previous history of drug uptake. Hence, an individual who prior to the last distribution of drugs had taken drugs twice, or three times or more, was about 2–3 times more likely to take drugs during the present round of MDA as compared to an individual who had not taken drugs before.

**Table 3:** Odds ratios (OR) for drug uptake in relation to personal characteristics in the combined interviewed adult study populations from the four study sites in Lindi and Morogoro Region.

Characteristic	No. individuals (% drug uptake)	Bivariate analysis		Multivariate analysis*		GEE**	
		OR (95 CI%)	P-value	OR (95 CI%)	P-value	OR (95 CI%)	P-value
Gender (n = 3279)							
Female	2106 (54.9)	1.00		-		-	
Male	1173 (55.8)	1.04 (0.90–1.20)	NS	-	-	-	-
Age group (n = 3279)							
15–29 years	1278 (49.6)	1.00		1.00		1.00	
30–49 years	1345 (58.0)	1.40 (1.20–1.64)	<0.001	1.46 (1.21–1.75)	<0.001	1.38 (1.13–1.70)	0.02
≥50 years	656 (60.4)	1.55 (1.28–1.87)	<0.001	1.63 (1.29–2.05)	<0.001	1.48 (1.15–1.19)	0.02
Level of education (n = 3006)							
Not completed primary school	495 (54.1)	1.00		-		-	
Completed primary school	2394 (57.4)	1.41 (0.93–2.13)	NS	-	-	-	-
Completed secondary school	117 (62.4)	1.41 (0.94–1.39)	NS	-	-	-	-
Wealth index (n = 2750)							
Low	658 (52.1)	1.00		1.00		1.00	
Medium	1552 (52.3)	1.01 (0.84–1.21)	NS	0.93 (0.76–1.15)	NS	0.91 (0.65–1.27)	NS
High	540 (63.5)	1.60 (1.27–2.02)	<0.001	0.98 (0.73–1.31)	NS	0.92 (0.73–1.14)	NS
Home ownership status (n = 3224)							
Own	1498 (53.8)	1.00		1.00		1.00	
Rented	430 (78.1)	3.07 (2.39–3.94)	<0.001	2.58 (1.70–3.91)	<0.001	2.59 (1.23–5.49)	0.013
Friends/relatives	948 (50.2)	0.87 (0.74–1.02)	NS	0.91 (0.75–1.11)	NS	0.89 (0.71–1.11)	NS
Employer	348 (45.7)	0.72 (0.57–0.91)	<0.01	1.08 (0.82–1.44)	NS	1.15 (0.83–1.59)	NS
Religion (n = 3260)							
Muslim	2366 (53.1)	1.00		1.00		1.00	
Christian	864 (60.9)	1.37 (1.71–1.61)	<0.001	0.97 (0.77–1.22)	NS	1.03 (0.79–1.33)	NS
Other	30 (43.3)	0.68 (0.33–1.40)	NS	0.74 (0.28–1.99)	NS	0.68 (0.21–2.19)	NS
Previous MDA drug uptake (n = 3168)							
Not before	1023 (38.8)	1.00		1.00		1.00	
Once	1037 (61.8)	2.55 (2.14–3.05)	<0.001	1.91 (1.55–2.35)	<0.001	1.52 (1.21–1.90)	<0.001
Twice	832 (63.1)	2.70 (2.23–3.26)	<0.001	2.27 (1.82–2,83)	<0.001	1.90 (1.49–2.42)	<0.001
Three or more times	276 (73.6)	4.39 (3.26–5.89)	<0.001	3.54 (2.53–4.95)	<0.001	2.84 (2.02–4.00)	<0.001

*) ORs have been adjusted for age, wealth index, home ownership status, religion, previous MDA drug uptake, and district.

**) Generalized Estimating Equations model, has been adjusted for the cluster effect of household in relation to drug uptake. The ORs are adjusted for age, wealth index, home ownership status, religion, previous MDA drug uptake, and district.

According to the interviewed adults who had taken the drugs, the vast majority of drug distributions (93.0%) had taken place in individual's homes, whereas 4.4% had been offered drugs from a central point in the target community (Table 4). The majority of drug distributors were reported to be community members (77.6%), but almost 10% of the interviewed individuals reported that they did not know the drug distributor. The vast majority of individuals who had taken drugs (95.0%) reported that their main reason for doing so was to protect themselves against LF.

**Table 4:** Answers to questions related to drug uptake among the interviewed adult study populations from the four study sites in Lindi and Morogoro Region who reported to have taken the drugs.

Question/answer	No. individuals (% of those who took the drugs)				
	Lindi Rural	Lindi Urban	Morogoro Rural	Morogoro Urban	Total
Where were you offered the drugs? (n = 1681)					
Brought to my home	280 (91.8)	424 (90.8)	235 (94.0)	624 (94,7)	1563 (93.0)
From a central point in our community	23 (7.5)	33 (7.1)	8 (3.1)	10 (1.5)	74 (4.4)
From a health facility	1 (0.3)	7 (1.5)	4 (1.6)	9 (1.4)	21 (1.2)
From my work place	0 (0.0)	0 (0.0)	0 (0.0)	10 (1.5)	10 (0.6)
From my school	0 (0.0)	2 (0.4)	1 (0.4)	3 (0.5)	6 (0.4)
Other	1 (0.3)	1 (0.2)	2 (0.8)	3 (0.5)	7 (0.4)
Who distributed the drugs? (n = 1784)					
Community members selected by community	287 (77.6)	337 (69.1)	229 (88.0)	531 (79.7)	1384 (77.6)
Health facility staff, community health worker, village leader	28 (7.5)	84 (17.2)	9 (3.5)	104 (15.6)	225 (12.6)
Don't know the distributor	55 (14.9)	67 (13.7)	22 (8.5)	31 (4.7)	175 (9.8)
Reason for taking the drugs (n = 1791)					
To protect myself against LF	354 (93.9)	465 (93.9)	248 (96.9)	635 (95.8)	1702 (95.0)
Other (e.g. instructed by leaders, because they are free)	23 (6.1)	30 (6.1)	8 (3.1)	28 (4.2)	89 (5.0)

### Reasons for not taking drugs

**Figure 2:**
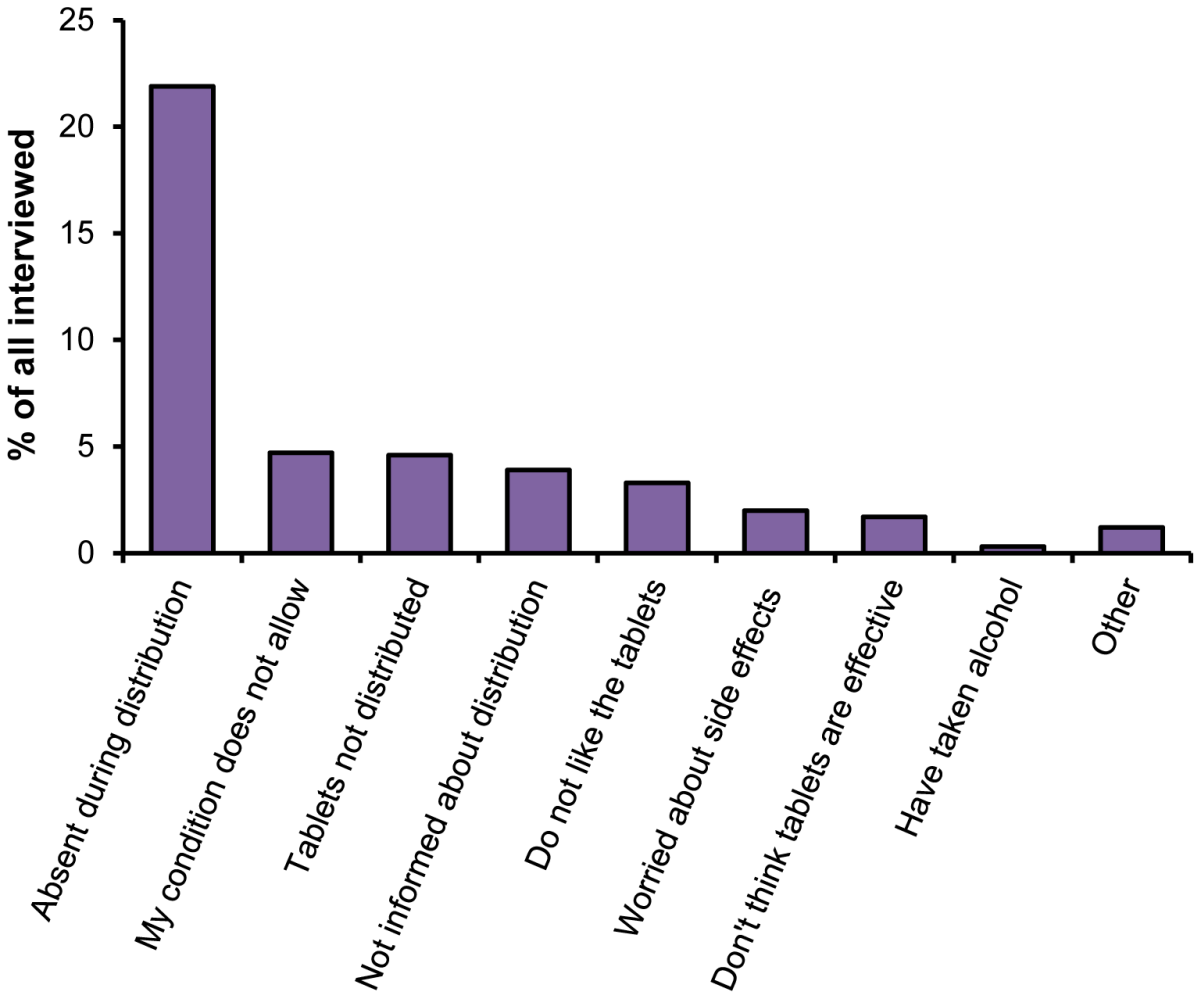
Reasons given for not taking the drugs. Shown for the combined interviewed adults (≥15 years) from the four study sites in Lindi and Morogoro Region. Expressed in percent of all interviewed individuals irrespective of whether they took the drugs or not (n = 3213).

Being absent from home during drug distribution was the most common reason given for not taking drugs and was reported by 50.2% of the individuals who did not take drugs during the last round of MDA (Table 5). This proportion corresponds to 21.9% of the total population not being at home when drug distributions were carried out (Figure 2). 10.6% of non-participants reported that tablets were never distributed to them, whereas 10.8% reported that they were excluded from treatment due to their condition (i.e. disease or pregnancy), and 9.0% reported that they were not informed about the drug distribution and therefore did not receive the drugs. Furthermore, 7.6% of the individuals who did not take drugs reported a general dislike for the drugs as the main reason for not taking them, 3.9% mentioned that they did not consider the drugs as being effective and 4.6% were worried about side effects.

**Table 5:** Reasons given for not taking the drugs among the interviewed adult study populations from the four study sites in Lindi and Morogoro Region who reported not to have taken the drugs (n = 1403).

Reason	Number individuals (% of those who did not take the drugs)				
	Lindi Rural	Lindi Urban	Morogoro Rural*	Morogoro Urban	Total
Absent from home during drug distribution	249 (59.6)	288 (49.3)	61 (37.4)	107 (45.0)	705 (50.2)
Drugs were not distributed	69 (16.5	39 (6.7)	-	41 (17.2)	149 (10.6)
Not allowed to take the drugs because of my condition	26 (6.2)	67 (11.5)	37 (22.7)	22 (9.2)	152 (10.8)
Not informed about distribution	42 (10.0)	31 (5.3)	21 (12.9)	32 (13.4)	126 (9.0)
Did not like the drugs	20 (4.8)	71 (12.2)	1 (0.6)	14 (5.9)	106 (7.6)
Worried about side effects	6 (1.4)	36 (6.2)	16 (9.8)	6 (2.5)	64 (4.6)
Don't think the drugs are effective	3 (0.7)	36 (6.2)	7 (4.3)	9 (3.8)	55 (3.9)
Had taken alcohol	1 (0.2)	5 (0.9)	1 (0.6)	1 (0.4)	8 (0.6)
Other reasons	2 (0.4)	11 (1.9)	19 (11.7)	6 (2.5)	38 (2.7)

*) The 374 individuals from Morogoro Rural who reported not to have been offered drugs were excluded from the study (see Methods).

## Discussion

The present study analysed factors influencing drug uptake in four districts of Tanzania shortly after implementation of MDA in 2011. When considering a recommended drug uptake rate of 65% or higher in order to interrupt transmission [10], the observed drug uptake rates in three of the four study districts were suboptimal. Lower than recommended drug uptake rates have similarly been reported from other LF control programmes in e.g. Sri Lanka [12], the Philippines [13], India [14], [16], [29], American Samoa [15], Kenya [17] and Ghana [18], as well as from previous studies on the LF control programme in Tanzania [27], [28], and may considerably prolong the time needed to reach the goal of LF transmission elimination [9]. It is of great importance to identify the barriers to optimal drug uptake rates in order to be able to improve on the rates and thereby secure successful LF control.

The overall questionnaire participation rate was high. A number of respondents subsequently had to be excluded from the analyses because the interviews were mistakenly performed prior to drug distribution (Morogoro Rural) or because of missing or contradicting data. However, it is unlikely that these exclusions have caused selection bias to the extent of significantly affecting the measured drug uptake rates and/or the results of the statistical analyses. Females accounted for a much higher proportion of the respondents than males at all study sites. This to some extent reflects the demographic profile in the study areas, where females outnumber males [30]. The gathered data were based on self-reported information. In order to minimize interviewers bias, including social desirability bias, comprehensive pilot testing and subsequent revisions of the questionnaires was performed prior to data collection, and the general impression was that the respondents were very eager to share their views and experiences with the researchers. Since the interviews were performed shorty after drug distribution it is likely that the influence of recall bias was also limited.

The higher levels of drug uptake in districts in Morogoro Region as compared to Lindi Region may be due to the more extensive previous experience with MDA activities in Morogoro Region, resulting in improved planning, more effective social mobilization and higher overall quality of drug distribution, including better timing and higher proportion of households covered by drug distributors. In Morogoro Urban the mobilization campaign moreover included the use of local radio and television stations, which offered their services to the programme free of charge. In this context, it was interesting that the highest drug uptake rate was observed in Morogoro Urban, in contrast to the common finding that drug uptake rates are lower in urban as compared to rural areas [12], [14], [16], [31], [32]. There was no clear overall difference in drug uptake rates between children and adults or between males and females, which may suggest that when one or several adult members in a household accept to take the drugs, the majority of the remaining household members will also do so. Similarly, no clear overall association was observed between drug uptake and individual educational level or household proxy indicators for socio-economic status. Assuming that lower educational or socio-economic levels are associated with a poorer knowledge about LF and its control, then the findings in the present study suggests that lack of this knowledge may not be a major barrier for drug uptake.

The GEE analysis showed that the major overall statistically significant predictors for drug uptake among the respondents were increasing age, living in a rented home and previous drug uptake in earlier rounds of MDA. After adjusting for other factors, including exposure to drug uptake in previous rounds of MDA, increasing age was associated with increasing levels of drug acceptance. This may reflect that older individuals are more motivated to take the drugs than the younger ones. To live in a rented home was also strongly associated with drug uptake. This trend was observed in both rural and urban districts of Morogoro Region, a region characterized by economic growth and a high influx of seasonal migrant workers, which has resulted in many houses and rooms being offered for rent. In contrast, very few individuals in Lindi Region lived in rented homes. The rented homes were primarily occupied by migrant workers originating from other parts of Tanzania, who were often more well off and better educated than the local population. Their perceptions and practices in relation to MDA apparently differed systematically from the rest of the population, thus leading to a higher level of drug uptake.

The strongest predictor for individuals to take drugs was previous history of drug uptake. Hence, individuals who had taken drugs three times or more prior to the present MDA were almost three times more likely to take drugs as compared to those who had not taken drugs before. This finding suggests that individuals who have already experienced the ancillary benefits of taking drugs, e.g. experiences with expulsion of *Ascaris* worms from the body [4], [11], and/or who gradually have obtained an increased level of acquaintance with and understanding of the principle of MDA are more motivated to take the drugs again during subsequent rounds of MDA. In this respect, a study in Kenya similarly showed that uptake of drugs was strongly associated with willingness to take drugs in future MDA rounds [17]. The findings of the present study moreover suggest that a small proportion of individuals for various reasons persistently refuse to take the drugs during MDA activities. These “systematic non-compliers” are a major problem to the control programmes as they serve as a continued source of infection for LF transmission in their community [11], [32], [33].

For the MDA providers in the control programme it is a major challenge to disseminate the message to the endemic population that the potential key LF health benefit is elimination of transmission rather than clearance of adult filarial worms in already infected individuals. Obviously, this complicated message is difficult to comprehend by the recipients. This was also seen in the present study where the most commonly reported reason for taking drugs was that treatment would protect the recipient against LF. This may also be the reason why the most commonly reported reason for taking drugs in the present study was that treatment would provide immediate personal protection of the recipient against LF. If this assumption is correct it is a concern that a partly incorrect perception of drug benefits is the main motivating factor for drug uptake.

The vast majority of respondents who took the drugs reported that drugs were offered to them in their homes. In this context, it is important to notice that being absent from home during time of drug distribution was the most common reason for not taking drugs, and accounted for more than 50% of all drug non-uptakes. This unfortunate occurrence was partly due to poor timing of drug distribution, e.g. in Lindi Rural drugs were distributed during harvest time – a period when many people are occupied in their farms. Another reason was poor communication from providers regarding the actual time of drug distribution, which resulted in many individuals waiting in vain for extended periods in their homes, where after they decided to leave before the drug distributor reached their part of the community (observed in a qualitative component of the present research project which will be reported elsewhere). The qualitative study component moreover revealed that some individuals, who already had made a decision not to take the drugs, deliberately left their homes at the time of drug distribution in order to avoid an encounter with the drug distributor, as also reported by others [27]. Other important provider-related reasons were given for not taking drugs, namely that the drugs were not distributed in their area, and that the recipients were not informed about the distribution. Similar observations have been reported from other studies [12], [14], [17], [18], [29], [31].

According to guidelines from the World Health Organization, a drug uptake rate of 65% or higher is required in order to eliminate transmission of LF [10]. The drug uptake rates observed in three of the four sites of the present study were lower or much lower than this. Unless steps are taken to increase drug uptake rates across endemic areas it is unlikely that the MDA programme will reach the target of elimination within reasonable time. In this respect, the findings of the present study provide useful insights for informed decisions on how to optimize drug delivery strategies. The findings strongly indicate that drug uptake relies more on easily modifiable provider-related factors than on individual perceptions and practices in the target population. Thus, limited investments in appropriate timing of drug distribution, dissemination of accurate information about the timing to recipients, and motivation of drug distributors to visit all households in the target areas (with repeat visits to households where inhabitants are not found at home during first visit) could potentially increase drug uptake considerably. This may be implemented by using a strategy that frequently monitors the programme performance and make appropriate adjustments. For this purpose, the Quality of Care model [34] developed by the World Health Organization provides a simple framework for engaging the various stakeholders, including representatives from different parts of the community, in the planning and implementation of an intervention in order to ensure a predefined level of quality. By using such systematic approach and by involving local decision makers, programme planners, drug distributors and community members, the MDA programmes targeting LF in Tanzania and elsewhere should be in a good position to increase drug uptake rates and thereby reach the target of global elimination of LF as a public health problem.

## Supporting Information

Table S1 Code sheet for MDA SPSS Database (adults).
(DOC)

Table S2 MDA SPSS Database for adults.
(SAV)

Table S3 Code sheet for MDA SPSS Database (children).
(DOC)

Table S4 MDA SPSS Database for children.
(SAV)
