# Supplementary material for: Factors Influencing Drug Uptake during Mass Drug Administration for Control of Lymphatic Filariasis in Rural and Urban Tanzania
Source: PLoS One. 2014 Oct 8;9(10):e109316. doi: 10.1371/journal.pone.0109316 (PMC4190414; doi:10.1371/journal.pone.0109316)
Supplement: Table S1 — Code sheet for MDA SPSS Database (adults). (DOC) [file pone.0109316.s001.doc]

CODEBOOK  Age [s] Q13ABUT [n] Q13BTAKE [n] Q13CNO [n] DATE [s] Q1REGIO [n] HouseHoldNo [n] Q3AATEN [n] Q3BLEVL [n] Q3CLEVL [n] Q4FAITH [n] Q6BOWNE [n] Q6CBICY [n] Q6CRADI [n] Q6CTV [n] Q6CMCYCL [n] Q6CAR [n] Q14ADRG [n] BUSE [n] Q14DNOT [n] Q15AMED [n]
Q15BWHO [n] BicyclePoint [n] RadioPoints [n] TVPoint [n] MotorcycklePoint [n] CarPoint [n] TotalItemScore [n] Q2SEX [n] AgeGroup [n] NewSchoolAttendance [n] NewHighestEduHousehold [n] HouseHoldItems [n] TakenDrugsBefore [n] NewDistrict [n] AgeGroup7 [n]
NewReligion [n] NewHouseOwnership [n] NewLevelEducation [n]
  /VARINFO POSITION LABEL TYPE FORMAT MEASURE ROLE VALUELABELS MISSING ATTRIBUTES
  /OPTIONS VARORDER=VARLIST SORT=ASCENDING MAXCATS=200
  /STATISTICS NONE.


Codebook


Notes	
Output Created	26-JUN-2014 12:47:19	
Comments		
Input	Data	/Users/dame/Desktop/MDA Data base for PLOS ONE (ADULTS).sav	
	Active Dataset	DataSet1	
	Filter	<none>	
	Weight	<none>	
	Split File	<none>	
	N of Rows in Working Data File	3279	
Syntax	CODEBOOK  Age [s] Q13ABUT [n] Q13BTAKE [n] Q13CNO [n] DATE [s] Q1REGIO [n] HouseHoldNo [n] Q3AATEN [n] Q3BLEVL [n] Q3CLEVL [n] Q4FAITH [n] Q6BOWNE [n] Q6CBICY [n] Q6CRADI [n] Q6CTV [n] Q6CMCYCL [n] Q6CAR [n] Q14ADRG [n] BUSE [n] Q14DNOT [n] Q15AMED [n]
Q15BWHO [n] BicyclePoint [n] RadioPoints [n] TVPoint [n] MotorcycklePoint [n] CarPoint [n] TotalItemScore [n] Q2SEX [n] AgeGroup [n] NewSchoolAttendance [n] NewHighestEduHousehold [n] HouseHoldItems [n] TakenDrugsBefore [n] NewDistrict [n] AgeGroup7 [n]
NewReligion [n] NewHouseOwnership [n] NewLevelEducation [n]
  /VARINFO POSITION LABEL TYPE FORMAT MEASURE ROLE VALUELABELS MISSING ATTRIBUTES
  /OPTIONS VARORDER=VARLIST SORT=ASCENDING MAXCATS=200
  /STATISTICS NONE.	
Resources	Processor Time	00:00:00.08	

Notes	
Resources	Elapsed Time	00:00:00.00	


[DataSet1] /Users/dame/Desktop/MDA Data base for PLOS ONE (ADULTS).sav


Age	
	Value	
Standard Attributes	Position	2	
	Label	Age in years (including individuals with YOB)	
	Type	Numeric	
	Format	F8	
	Measurement	Scale	
	Role	Input	


Q13ABUT	
	Value	
Standard Attributes	Position	3	
	Label	13a.Ever taken MDA tablets this year            ..	
	Type	Numeric	
	Format	F1	
	Measurement	Nominal	
	Role	Input	
Valid Values	1	Yes	
	2	No	


Q13BTAKE	
	Value	
Standard Attributes	Position	4	
	Label	13b.If Yes, why did you use the tablets         ..	
	Type	Numeric	
	Format	F1	
	Measurement	Nominal	
	Role	Input	
Valid Values	1	Prevent against LF	
	2	Drugs have other benefits	
	3	Because they are free	
	4	Instructed by leaders	
	5	Many people take them	
	7	No reason	
	8	No answer	


Q13CNO	
	Value	
Standard Attributes	Position	5	
	Label	13c.If no, why didn't you use the tablets       ..	
	Type	Numeric	
	Format	F2	
	Measurement	Nominal	
	Role	Input	
Valid Values	1	Absent	
	2	The tablets were not distributed	
	3	Not allowed to take tablets because of my condition	
	4	I did not take tablets because I took alcohol	
	5	Not informed about distribution	
	6	I did not like to take the tablets	
	7	Fear of side effects	
	8	I don't think the tablets are effective	
	10	I don't remember	
	11	Other reasons	


DATE	
	Value	
Standard Attributes	Position	6	
	Label	Date: Date	
	Type	Numeric	
	Format	DATE11	
	Measurement	Scale	
	Role	Input	


Q1REGIO	
	Value	
Standard Attributes	Position	7	
	Label	1. Name of Region:         Q1regio	
	Type	String	
	Format	A19	
	Measurement	Nominal	
	Role	Input	


HouseHoldNo	
	Value	
Standard Attributes	Position	8	
	Label	= VillageNo + KAYA	
	Type	Numeric	
	Format	F10	
	Measurement	Nominal	
	Role	Input	


Q3AATEN	
	Value	
Standard Attributes	Position	9	
	Label	3a. Have attend school                  Q3Aaten	
	Type	Numeric	
	Format	F1	
	Measurement	Nominal	
	Role	Input	
Valid Values	1	Yes	
	2	No	
	3	No answer	


Q3BLEVL	
	Value	
Standard Attributes	Position	10	
	Label	3b. Level of education                  Q3Blevl	
	Type	Numeric	
	Format	F1	
	Measurement	Nominal	
	Role	Input	
Valid Values	1	No education	
	2	STD 0-4 yrs	
	3	primary (std5-7yrs)	
	4	secondary (form1-4)	
	5	higher education (above form4)	
	6	don't know	


Q3CLEVL	
	Value	
Standard Attributes	Position	11	
	Label	3c. Level of education in the household Q3Clevl	
	Type	Numeric	
	Format	F1	
	Measurement	Nominal	
	Role	Input	
Valid Values	1	no education	
	2	std 0-4yrs	
	3	primary (std5-7yrs)	
	4	secondary (form1-4)	
	5	higher education (above form4)	
	6	don't know	


Q4FAITH	
	Value	
Standard Attributes	Position	12	
	Label	4.  Religious faith follow              Q4faith	
	Type	Numeric	
	Format	F1	
	Measurement	Nominal	
	Role	Input	
Valid Values	1	christianty	
	2	islam	
	3	traditional	
	4	others	
	5	no belief	
	6	don't know	
	7	no answer	


Q6BOWNE	
	Value	
Standard Attributes	Position	13	
	Label	6b. Owner of this house/apartment       Q6Bowne	
	Type	Numeric	
	Format	F1	
	Measurement	Nominal	
	Role	Input	
Valid Values	1	own house	
	2	relatives house/apartment	
	3	friend house/apartment	
	4	rent house/apartment	
	5	office company house/apartment	
	6	don't know	


Q6CBICY	
	Value	
Standard Attributes	Position	14	
	Label	(i)Bicycle       Q6CBicy	
	Type	Numeric	
	Format	F1	
	Measurement	Nominal	
	Role	Input	
Valid Values	1	yes	
	2	No	
	3	no answer	


Q6CRADI	
	Value	
Standard Attributes	Position	15	
	Label	(ii)Radio        q6CRadi	
	Type	Numeric	
	Format	F1	
	Measurement	Nominal	
	Role	Input	
Valid Values	1	yes	
	2	no	
	3	no answer	


Q6CTV	
	Value	
Standard Attributes	Position	16	
	Label	(iii)TV          Q6CTV	
	Type	Numeric	
	Format	F1	
	Measurement	Nominal	
	Role	Input	
Valid Values	1	yes	
	2	no	
	3	no answer	


Q6CMCYCL	
	Value	
Standard Attributes	Position	17	
	Label	(iv)Motorcycle   Q6CMCycl	
	Type	Numeric	
	Format	F1	
	Measurement	Nominal	
	Role	Input	
Valid Values	1	yes	
	2	no	
	3	no answer	


Q6CAR	
	Value	
Standard Attributes	Position	18	
	Label	(v)Car           Q6Car	
	Type	Numeric	
	Format	F1	
	Measurement	Nominal	
	Role	Input	
Valid Values	1	yes	
	2	no	
	3	no answer	


Q14ADRG	
	Value	
Standard Attributes	Position	19	
	Label	14a.Do you have ingested these drugs before this..	
	Type	Numeric	
	Format	F1	
	Measurement	Nominal	
	Role	Input	
Valid Values	1	Yes	
	2	No	
	3	No answer	
	4	Dont know	


BUSE	
	Value	
Standard Attributes	Position	20	
	Label	14b.Before the allocation of this year was how o..	
	Type	Numeric	
	Format	F1	
	Measurement	Nominal	
	Role	Input	
Valid Values	1	One time	
	2	Two times	
	3	Three times	
	4	More than once	
	5	I don't remember	
	6	No answer	


Q14DNOT	
	Value	
Standard Attributes	Position	21	
	Label	14d.Why not use these drugs before this year	
	Type	Numeric	
	Format	F2	
	Measurement	Nominal	
	Role	Input	
Valid Values	1	Absence	
	2	Tablets not distributed	
	3	Not allowed to take tablets because of my condition	
	4	I did not take tablets because I took alcohol	
	5	Not informed about distribution	
	6	I did not like the tablets	
	7	Fear of side effects	
	8	I did not have a disease	
	9	I dont think the tablets are effective	
	10	I dont remember	
	11	Others	


Q15AMED	
	Value	
Standard Attributes	Position	22	
	Label	15a.Here is the village where you were given med..	
	Type	Numeric	
	Format	F1	
	Measurement	Nominal	
	Role	Input	
Valid Values	1	The tablets were brought to my home	
	2	The tablets were given at a central place	
	3	The tablets were given in a health facility	
	4	The tablets were brought to my work place	
	5	The tablets were brought to my school	
	6	Others	


Q15BWHO	
	Value	
Standard Attributes	Position	23	
	Label	15b.Who distribute the drugs                    ..	
	Type	Numeric	
	Format	F1	
	Measurement	Nominal	
	Role	Input	
Valid Values	1	Staff person at the health facility	
	2	Community health worker	
	3	Community member selected to distribute drugs	
	4	Village leader	
	5	Others	
	6	I dont know	


BicyclePoint	
	Value	
Standard Attributes	Position	24	
	Label	BicyclePoint (Yes = 4 points; No = 0 points)	
	Type	Numeric	
	Format	F8	
	Measurement	Nominal	
	Role	Input	


RadioPoints	
	Value	
Standard Attributes	Position	25	
	Label	RadioPoint (Yes = 2 points; No = 0 points)	
	Type	Numeric	
	Format	F8	
	Measurement	Nominal	
	Role	Input	


TVPoint	
	Value	
Standard Attributes	Position	26	
	Label	TVPoint (Yes = 6 points; No = 0 points)	
	Type	Numeric	
	Format	F8	
	Measurement	Nominal	
	Role	Input	


MotorcycklePoint	
	Value	
Standard Attributes	Position	27	
	Label	MotorcycklePoint (Yes = 8 points; No = 0 points)	
	Type	Numeric	
	Format	F8	
	Measurement	Nominal	
	Role	Input	


CarPoint	
	Value	
Standard Attributes	Position	28	
	Label	CarPoint (Yes = 10 points; No = 0 points)	
	Type	Numeric	
	Format	F8	
	Measurement	Nominal	
	Role	Input	


TotalItemScore	
	Value	
Standard Attributes	Position	29	
	Label	Total sum of 5 items (Tbike, radio, TV, MC. car)	
	Type	Numeric	
	Format	F8	
	Measurement	Nominal	
	Role	Input	


Q2SEX	
	Value	
Standard Attributes	Position	30	
	Label	2.  Sex:                                Q2Sex:	
	Type	Numeric	
	Format	F1	
	Measurement	Nominal	
	Role	Input	
Valid Values	1	Male	
	2	Female	


AgeGroup	
	Value	
Standard Attributes	Position	31	
	Label	AgeGroup	
	Type	Numeric	
	Format	F8	
	Measurement	Nominal	
	Role	Input	
Valid Values	1	>= 50 yrs	
	2	30-49 yrs	
	3	15-29 yrs	


NewSchoolAttendance	
	Value	
Standard Attributes	Position	32	
	Label	School attendance	
	Type	Numeric	
	Format	F8	
	Measurement	Nominal	
	Role	Input	
Valid Values	1	Yes	
	2	No	


NewHighestEduHousehold	
	Value	
Standard Attributes	Position	33	
	Label	Highest education in household	
	Type	Numeric	
	Format	F8	
	Measurement	Nominal	
	Role	Input	
Valid Values	1	Secondary education or higher	
	2	Primary education	
	3	No education	


HouseHoldItems	
	Value	
Standard Attributes	Position	34	
	Label	HouseHold Items level	
	Type	Numeric	
	Format	F8	
	Measurement	Nominal	
	Role	Input	
Valid Values	1	Highest (sum >= 7)	
	2	Medium (sum = 2-6 points)	
	3	Lowest (sum = 0 points)	


TakenDrugsBefore	
	Value	
Standard Attributes	Position	35	
	Label	Previous drug taking	
	Type	Numeric	
	Format	F8	
	Measurement	Nominal	
	Role	Input	
Valid Values	1	Three times or more before	
	2	Two times before	
	3	Once before	
	4	Not before	


NewDistrict	
	Value	
Standard Attributes	Position	36	
	Label	NewDistrict	
	Type	Numeric	
	Format	F8	
	Measurement	Nominal	
	Role	Input	
Valid Values	1	Lindi rural	
	2	Lindi urban	
	3	Morogoro rural	
	4	Morogoro urban	


AgeGroup7	
	Value	
Standard Attributes	Position	37	
	Label	Age Groups (7 groups)	
	Type	Numeric	
	Format	F8	
	Measurement	Nominal	
	Role	Input	
Valid Values	1	15-19 years	
	2	20-29 years	
	3	30-39 years	
	4	40-49 years	
	5	50-59 years	
	6	60-69 years	
	7	>= 70 years	


NewReligion	
	Value	
Standard Attributes	Position	38	
	Label	Religion 3 groups	
	Type	Numeric	
	Format	F8	
	Measurement	Nominal	
	Role	Input	
Valid Values	1	Other + traditionel	
	2	Christian	
	3	Islam	


NewHouseOwnership	
	Value	
Standard Attributes	Position	39	
	Label	NewHouseOwnership	
	Type	Numeric	
	Format	F8	
	Measurement	Nominal	
	Role	Input	
Valid Values	1	Company	
	2	Rented	
	3	Friends/relatives	
	4	Own	


NewLevelEducation	
	Value	
Standard Attributes	Position	40	
	Label	Levels of education (3)	
	Type	Numeric	
	Format	F8	
	Measurement	Nominal	
	Role	Input	
Valid Values	1	Secondary or higher	
	2	Primary education	
	3	No education	
