# Supplementary material for: Factors Influencing Drug Uptake during Mass Drug Administration for Control of Lymphatic Filariasis in Rural and Urban Tanzania
Source: PLoS One. 2014 Oct 8;9(10):e109316. doi: 10.1371/journal.pone.0109316 (PMC4190414; doi:10.1371/journal.pone.0109316)
Supplement: Table S3 — Code sheet for MDA SPSS Database (children). (DOC) [file pone.0109316.s003.doc]

SAVE OUTFILE='/Users/dame/Documents/William 2014/TIL PLOS ONE SPSS DATA + CODESHEETS/MDA '+
    'database (n=1942, CHILDREN).sav'
  /COMPRESSED.
CODEBOOK  District [n] TAKEN [n] AGE [s] GENDER [n]
  /VARINFO POSITION LABEL TYPE FORMAT MEASURE ROLE VALUELABELS MISSING ATTRIBUTES
  /OPTIONS VARORDER=VARLIST SORT=ASCENDING MAXCATS=200
  /STATISTICS COUNT PERCENT MEAN STDDEV QUARTILES.


Codebook


Notes	
Output Created	26-JUN-2014 13:31:03	
Comments		
Input	Data	/Users/dame/Documents/William 2014/TIL PLOS ONE SPSS DATA + CODESHEETS/MDA database (n=1942, CHILDREN).sav	
	Active Dataset	DataSet1	
	Filter	<none>	
	Weight	<none>	
	Split File	<none>	
	N of Rows in Working Data File	1942	
Syntax	CODEBOOK  District [n] TAKEN [n] AGE [s] GENDER [n]
  /VARINFO POSITION LABEL TYPE FORMAT MEASURE ROLE VALUELABELS MISSING ATTRIBUTES
  /OPTIONS VARORDER=VARLIST SORT=ASCENDING MAXCATS=200
  /STATISTICS COUNT PERCENT MEAN STDDEV QUARTILES.	
Resources	Processor Time	00:00:00.02	
	Elapsed Time	00:00:00.00	


[DataSet1] /Users/dame/Documents/William 2014/TIL PLOS ONE SPSS DATA + CODESHEETS/MDA database (n=1942, CHILDREN).sav


District	
	Value	Count	Percent	
Standard Attributes	Position	1			
	Label	NewDistrict			
	Type	Numeric			
	Format	F8			
	Measurement	Nominal			
	Role	Input			
Valid Values	1	Lindi rural	455	23.4%	
	2	Lindi urban	679	35.0%	
	3	Morogoro rural	259	13.3%	
	4	Morogoro urban	549	28.3%	


TAKEN	
	Value	Count	Percent	
Standard Attributes	Position	2			
	Label	Tablets taken			
	Type	Numeric			
	Format	F1			
	Measurement	Nominal			
	Role	Input			
Valid Values	1	Yes	1065	54.8%	
	2	No	877	45.2%	


AGE	
	Value	
Standard Attributes	Position	3	
	Label	Age of child	
	Type	Numeric	
	Format	F2	
	Measurement	Scale	
	Role	Input	
N	Valid	1942	
	Missing	0	
Central Tendency and Dispersion	Mean	9.26	
	Standard Deviation	2.987	
	Percentile 25	7.00	
	Percentile 50	9.00	
	Percentile 75	12.00	


GENDER	
	Value	Count	Percent	
Standard Attributes	Position	4			
	Label	Gender of child			
	Type	Numeric			
	Format	F1			
	Measurement	Nominal			
	Role	Input			
Valid Values	1	Male	1020	52.5%	
	2	Female	922	47.5%	
